# Silver nanoparticles produced from *Cedecea* sp. exhibit antibiofilm activity and remarkable stability

**DOI:** 10.1038/s41598-021-92006-4

**Published:** 2021-06-16

**Authors:** Priyanka Singh, Santosh Pandit, Carsten Jers, Abhayraj S. Joshi, Jørgen Garnæs, Ivan Mijakovic

**Affiliations:** 1 The Novo Nordisk Foundation, Center for Biosustainability, Technical University of Denmark, 2800 Lyngby, Denmark; 2 Systems and Synthetic Biology Division, Department of Biology and Biological Engineering, Chalmers University of Technology, 412 96 Gothenburg, Sweden; 3 Danish Fundamental Metrology, Kogle Allé 5, 2970 Hørsholm, Denmark

**Keywords:** Microbiology, Pathogenesis, Nanoscience and technology

## Abstract

With multidrug-resistant bacterial pathogens on the rise, there is a strong research focus on alternative antibacterial treatments that could replace or complement classical antibiotics. Metallic nanoparticles, and in particular silver nanoparticles (AgNPs), have been shown to kill bacterial biofilms effectively, but their chemical synthesis often involves environmentally unfriendly by-products. Recent studies have shown that microbial and plant extracts can be used for the environmentally friendly synthesis of AgNPs. Herein we report a procedure for producing AgNPs using a putative *Cedecea* sp. strain isolated from soil. The isolated bacterial strain showed a remarkable potential for producing spherical, crystalline and stable AgNPs characterized by UV–visible spectroscopy, transmission electron microscopy, dynamic light scattering, and Fourier transform infrared spectroscopy. The concentration of produced nanoparticles was 1.31 µg/µl with a negative surface charge of − 15.3 mV and nanoparticles size ranging from 10–40 nm. The AgNPs was tested against four pathogenic microorganisms *S. epidermidis*, *S. aureus*, *E. coli* and *P. aeruginosa*. The nanoparticles exhibited strong minimum inhibitory concentration (MIC) values of 12.5 and 6.25 µg/µl and minimum bactericidal concentration (MBC) values of 12.5 and 12.5 µg/mL against *E. coli* and *P. aeruginosa*, respectively. One distinguishing feature of AgNPs produced by *Cedecea* sp. extracts is their extreme stability. Inductively coupled plasma mass spectrometry and thermogravimetric analysis demonstrated that the produced AgNPs are stable for periods exceeding one year. This means that their strong antibacterial effects, demonstrated against *E. coli* and *P. aeruginosa* biofilms, can be expected to persist during extended periods.

## Introduction

Microbial infections and microbes' resistance to commonly used antibiotics are the leading causes of death worldwide, and this has a tremendous impact on public health and the health economy^1^. Most pathogenic bacterial species tend to aggregate, adhere, multiply and form a 3-dimensional (3D) complex network on biotic or abiotic surfaces. This complex 3D multilayered network is known as biofilm^2^. Biofilms hold dormant bacteria, which can move to another surface to form a new biofilm after maturation. The extracellular components of biofilms: polysaccharides, nucleic acids, proteins and lipids, interact with each other and the embedded bacterial cells^3^. These extracellular components perform many functions such as adhesion, protection, nutrient and metabolite resourcing, and they confer resistance to the unfavorable surrounding environment and antimicrobial agents^4^. Biofilms are a particularly effective shield against antibiotics, slowing down their penetration, provoking their enzymatic degradation, and providing shielded bacterial cells with an opportunity to develop resistance via genetic changes^5^. Biofilm components have been recognized as excellent targets for antimicrobial therapy^4,6^ since biofilms' disruption would render bacterial cells more vulnerable to antibiotics and the immune system. To overcome the limitation of antibiotics-based treatment of biofilms, new antimicrobial therapies are being developed. These include developing small molecules, immunomodulators, anti-virulence agents, antibodies, vaccines and metallic nanoparticles^7,8^.

Noble metals have been known for their antimicrobial properties throughout human history. Since ancient times, silver salts, metallic silver, and silver compounds have been successfully employed for preventing microbial growth^9^. Advances in nanotechnology made it possible to synthesize silver nanoparticles (AgNPs), which, due to their large surface area to volume ratio, exhibit remarkable antibacterial properties against a range of microbial pathogens^10^. To date, many reports have been published that showed the action of AgNPs against planktonic bacterial cells as well as biofilms of multidrug-resistant bacteria^11,12^.

**Figure 1:**
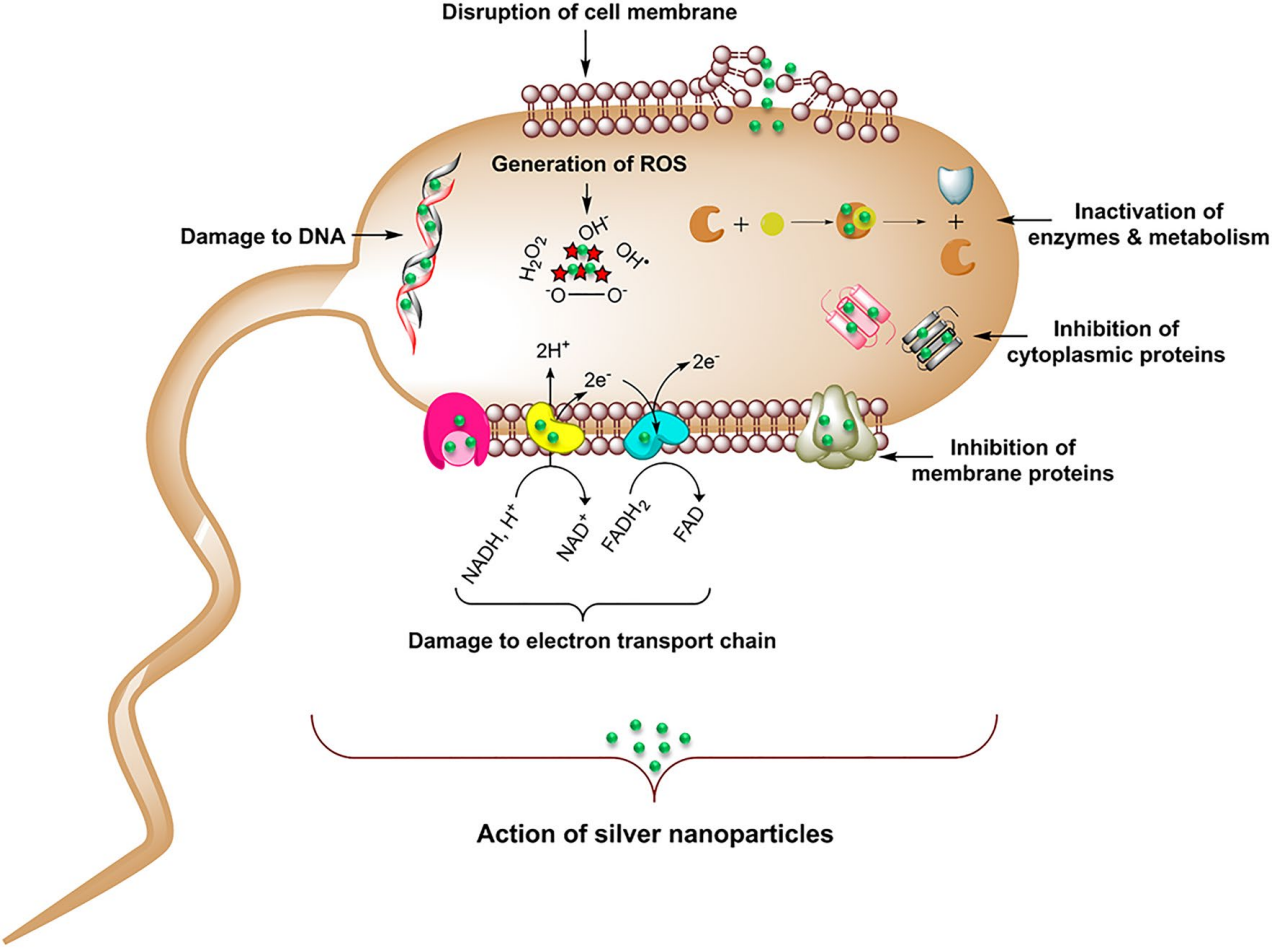
Schematic representation of the proposed mode of action for produced silver nanoparticles (AgNPs) on bacteria cell apoptosis.

AgNPs offer several advantages for antibiofilm applications, such as the high surface area to volume ratio, inert nature, tunable physical properties such as shape and size, biocompatibility, and demonstrated bacteriostatic or bactericidal properties at very low concentrations^13^. The antimicrobial action comes directly from AgNPs, as well as from silver ions (Ag^+^) formed from their dissolution. Both AgNPs and Ag^+^ ions can interact with multiple components of planktonic bacterial cells and biofilm components. Through these interactions, they interfere with bacterial metabolism and hamper outer cellular functions^14^. Overall antimicrobial effect of AgNPs comes from a combination of cell wall destruction, structural protein destabilization, membrane protein inactivation, enzyme inactivation, inhibition of electron transport chain (ETC), damage to nucleic acids and oxidative stress elicited via reactive oxygen species (ROS) generation (Fig. 1)^15^. Thus, AgNPs have become a promising choice for antimicrobial therapy^16^.

AgNPs can be synthesized using various methods that can be categorized as 'top-down' or 'bottom-up' approaches. The bottom-up approach i.e. 'building nanoparticles from silver atoms in an aqueous solution' is the most widely used method. Conventional AgNPs synthesis methods use hazardous chemicals and solvents and specialized equipment, which make them very costly and cumbersome. In addition, some of those methods generate toxic by-products^14^. However, the so-called "biosynthesis" approach for AgNPs formation offers an environmentally friendly and low-cost alternative^17,18^. Recently, several reports have been published describing ecologically friendly production of AgNPs using extracts of bacterial and plant cells as nano-factories with high potential for scaling up^19,20^. Various biomolecules of plant and bacterial origin in these nano-factories (proteins, enzymes, vitamins, polysaccharides etc.) are known to interact with the produced AgNPs, enhancing their biocompatibility and stability^21^. In particular, sugars, polyphenols, acids, proteins, terpenoids and polysaccharides present in biological nano-factories strongly contribute to bio-reduction of silver ions to atomic silver, and thereby their deposition into AgNPs. These biological compounds cover the AgNPs' surface, which in turn enhances their stability and biocompatibility ^22,23^.

Following the "biosynthesis" approach for NP synthesis, in the present study we examined for the first time the capacity of bacteria from the *Cedecea* family to support the production of AgNPs with antibiofilm potential. We have demonstrated that a *Cedecea* sp. environmental isolate can serve as a source of AgNPs with excellent antibacterial properties, comparable to other biogenic AgNPs reported in the literature. A distinguishing feature of AgNPs produced extracellularly by *Cedecea* sp. is their extreme physical stability, leading to undiminished antibacterial effects for periods exceeding one year. Moreover, the extracellular synthesis confers an important advantage over intracellular synthesis by bacteria since it requires no additional steps for NPs purification^24^, such as ultra-sonication, water or solvent washing. This makes the AgNPs production process more feasible and economical.

## Materials and methods

### Materials

Silver nitrate (AgNO_3_), tryptic soya agar (TSA), and tryptic soya broth (TSB) were purchased from Sigma-Aldrich Chemicals, St. Louis, USA.

### Isolation and 16S rDNA gene sequencing

Soil sample was collected in sterile poly bags from Technical University of Denmark (DTU) field, Lyngby, Denmark. For isolation, a previously reported methodology was followed^25^. Briefly, the individual colonies were isolated by using the serial dilution technique on TSA plates. The isolated colonies were further screened out by allowing the strains to grow on TSA plate with 1 mM AgNO_3_ at 37 °C for 24 h. Molecular identification of the isolated bacteria was performed using 16S rDNA amplification and sequencing^26^. Genomic DNA was isolated using the DNeasy Blood and Tissue Kit (Qiagen) and used as a template for PCR with the universal primers 27 F (5′-AGAGTTTGATCMTGGCTCAG-3′) and 1492 R (3′-TACGGYTACCTTGTTACGACTT-5′)^27^. Eurofins Genomics (Ebensburg, Germany) sequenced the PCR product and the sequence was analyzed using the NCBI BLAST homepage against the reference sequence database.

### Synthesis and purification of AgNPs

**Figure 2:**
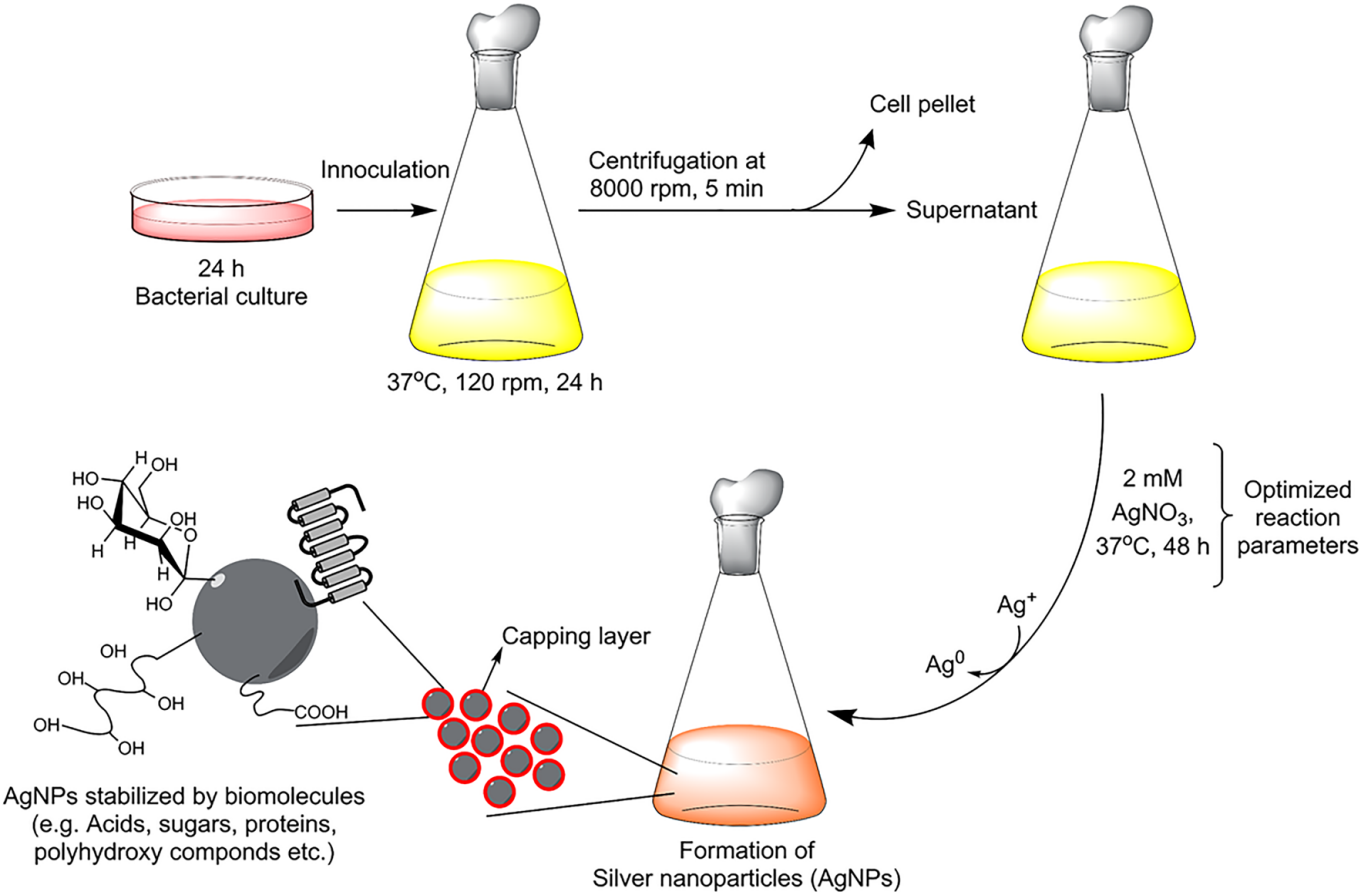
Schematics showing the stepwise procedure for AgNPs production from isolated *Cedecea* sp.

"Biosynthesis" synthesis of AgNPs was done as reported previously^25^. Briefly, 100 ml of overnight bacteria culture was spun down 8000 rpm for 5 min to get cell-free supernatant. 2 mM of AgNO_3_ was added to this cell-free supernatant and incubated further at 37 °C for 24–48 h. The salt mix supernatant (reaction mixture) was monitored continuously for nanoparticle synthesis, by visual inspection and by recording UV–Vis spectra. AgNPs were purified by centrifugation at 14,000 rpm for 10 min. After centrifugation, the supernatant was discarded and AgNPs were washed 3 times with sterile water and obtained in the precipitate form. This residue was suspended again into sterile water and used for all experiments. Figure 2 showed the schematic illustration of nanoparticles formation from the supernatant of *Cedecea* sp.

### Analytical characterization of AgNPs

The UV–Vis spectrum of produced AgNPs was obtained using 6705 UV–Vis spectrophotometer, JENWAY, by scanning it in the range of 300–700 nm. The optimization studies for AgNPs production were also conducted using UV–Vis spectroscopy^28^. To analyze the internal morphology, composition and crystallographic information of produced AgNPs, transmission electron microscopy (TEM) was carried out using the FEI Tecnai T20 G2 instrument, operated at an acceleration voltage of 200 kV. AgNPs sample for TEM was prepared by spotting the pure NPs solution onto carbon coated cupper grid, followed by complete air-drying prior to examination in the microscope. The nanoparticle size distribution and the surface charge was studied by Dynamic light scattering (DLS) (Zetasizer Nano ZS, Chuo-ku Kobe-shi, Japan), following the previously reported methodology^28^. Atomic force microscopy (AFM) (Park NX20 from www.parkafm.com) measurements were carried out in intermittent contact mode using standard probes of single crystal highly doped silicon with a radius of curvature of less than 30 nm (SuperSharpSilicon™ Non-Contact AFM probes from Nanosensors). The standard uncertainty *u*(*d*) of the measured diameters are *u*(*d*) < 0.05 d. The FTIR measurements were carried out using Nicolet iS50 (Thermo Fisher Scientific, Waltham, MA, USA) by scanning the air-dried purified AgNPs and cell's supernatant over the range of 4000–450 cm^−1^ at a resolution of 4 cm^−1^. The recorded spectra recorded were plotted as transmittance (%) versus wavenumber (cm^−1^)^15^.

For size fractionation and quantification of nanoparticles sp-ICP-MS (NexION 350D; PerkinElmer Inc., Waltham, MA, USA) was used in similar conditions as reported previously^29^. Nanoparticle stability was studied by keeping the NPs solution in ambient conditions, such as different pH, time, temperature, and bacteriological media such as TSB and Luria broth (LB) for indicated amounts of time. The UV–Vis spectra were measured before and after the incubation period. The NPs were characterized by sp-ICP-MS, and UV–Vis for analyzing stability and spectra were measured before and after the incubation period. The thermal stability of nanoparticles was determined by thermogravimetric analysis (TGA), for which the nanoparticles in powder form was performed on a Discovery TGA (TA Instruments, New Castle, DE, USA). For analyzing TGA, the nanoparticles were placed in an alumina pan and heated from 20 to 700 °C at a ramping time of 10 °C/min."

### Bacterial strains and culture media

The antimicrobial activity of AgNPs was evaluated against four pathogens, comprising both Gram-positive and Gram-negative bacteria: *Escherichia coli* UTI 89, *Pseudomonas aeruginosa* PAO1, *Staphylococcus epidermidis* ATCC 35,984, and *Staphylococcus aureus* CCUG 10,778. LB medium was used for *E. coli* and *P. aeruginosa*, and TSB was used for *S. epidermidis* and *S. aureus*.

### Minimum inhibitory concentration (MIC) and minimum bactericidal concentration (MBC) of AgNPs

The bacteriostatic and bactericidal activity of AgNPs was evaluated by measuring MIC and MBC, respectively, using the following microdilution assay. The bacteria cultures were grown overnight and diluted to approximately 1–2 × 10^5^ colony forming units (CFU)/mL. Then, the AgNPs were added in concentrations ranging from 1.56 to 50 µg/mL, in twofold dilution series. Samples were incubated at 37 °C, for 24 h. After 24 h, bacterial growth was measured by recording OD_550_ for all samples. The MIC was defined as the lowest concentration of AgNPs, which inhibited the bacterial growth, measured as OD_550_.

The MBC value was defined as the lowest concentration of AgNPs that was required to kill the bacterial strain. For measuring MBC, 100 µL of the mixtures as mentioned above was streaked on agar plates and incubated at 37 °C overnight. This was followed by CFU counting to estimate the survival of bacterial cells^25^.

### Biofilm inhibition assay

For biofilm inhibition assay, the overnight grew bacterial culture was diluted to the final concentration of 1–2 × 10^6^ CFU/mL. 200 µL of this suspension was added to a 96-well plate and incubated for 5 h at 37 °C. After the incubation period, different concentrations of AgNPs were added in the suspension and incubated further for 19 h at 37 °C. After 24 h (in total), the static biofilm formed on the wall of 96 well plates, the medium was carefully removed and the biofilm was washed twice with sterile water. The biofilms were stained with 0.1% crystal violet for 20 min, washed with water and dried at room temperature for 1 h. Finally, absolute ethanol (200 µL) was added to the stained biofilms, and the samples were agitated vigorously for 15 min to extract the stain. The absorbance of extracted crystal violet was measured at 590 nm^28^.

### Bacterial viability after AgNPs exposure

Three different methods analyzed the biofilm inhibition activity of AgNPs: (1) by checking the bacterial viability, (2) by observing the cells under the scanning electron microscope (SEM), and (3) by live/dead staining assay^25,28^. The inoculum of 2–5 × 10^6^ CFU/mL was obtained by diluting overnight cultures of *E. coli* and *P. aeruginosa and* loading the samples on top of a 15 mm cover glass. The cover glass was further incubated at 37 °C for 24 h, which allowed for biofilm formation. After 24 h, old culture medium was replaced with fresh medium, containing sterile water (negative control) or 1 × MBC, 2 × MBC, 4 × MBC, or 8 × MBC of AgNPs. Sample were incubated for further 24 h. Then the biofilms were homogenized for 20 s in 0.89% NaCl. The obtained bacterial samples were serially diluted and plated on LB agar plates. Next, the plates were incubated overnight at 37 °C, followed by CFU counting. For live/dead cells staining, samples were stained for 20 min with a mixture of 6.0 μM SYTO 9 and 30 μM KI from Live/Dead BacLight Viability kit L13152, (Invitrogen, Molecular Probes, Inc. Eugene, Oregon, USA). Fluorescence microscopy were collected using a Zeiss fluorescence microscope (Axio Imager.Z2m Carl Zeiss, Zena, Germany). Further, control and AgNPs-treated biofilms were examined by SEM (Supra 60 VP microscope, Carl Zeiss AG)^28^.

### Statistical analysis

All experiments were done in three biological and two technical replicates and data are presented as the mean ± standard deviation (SD). The intergroup differences were estimated by one-way analysis of variance (ANOVA), followed by a post hoc multiple comparisons (Tukey test). Values were considered statistically significant at *p* < 0.05.^15^.

## Results

### Molecular characterization of the isolated environmental strain

The environmental isolate has been selected based on prescreening of individual colonies on TSA plates supplemented with 1 mM AgNO_3_, which means the strain was able to tolerate silver salt at the desired conc. to produce nanoparticles. The sequencing of 16S rDNA from our bacterial strain isolated from soil indicated 98.17% identity with *Cedecea lapagei* strain DSM 4587. The isolated strain sequence number is submitted to NCBI with GenBank: MT524486.1. *Cedecea lapagei* is reported to be a Gram-negative, non-sporulating, motile, non/encapsulated, rod-shaped bacilli, which belongs to the *Enterobacteriaceae* family. Various species of this genus have been reported to be isolated from various clinical specimens including sputum, urine, cutaneous and oral ulcers etc^30–32^. Pending further molecular characterization, we will refer to this strain as *Cedecea* sp.

### Cell-free supernatant of *Cedecea* sp. is a suitable medium for the synthesis of AgNPs

**Figure 3:**
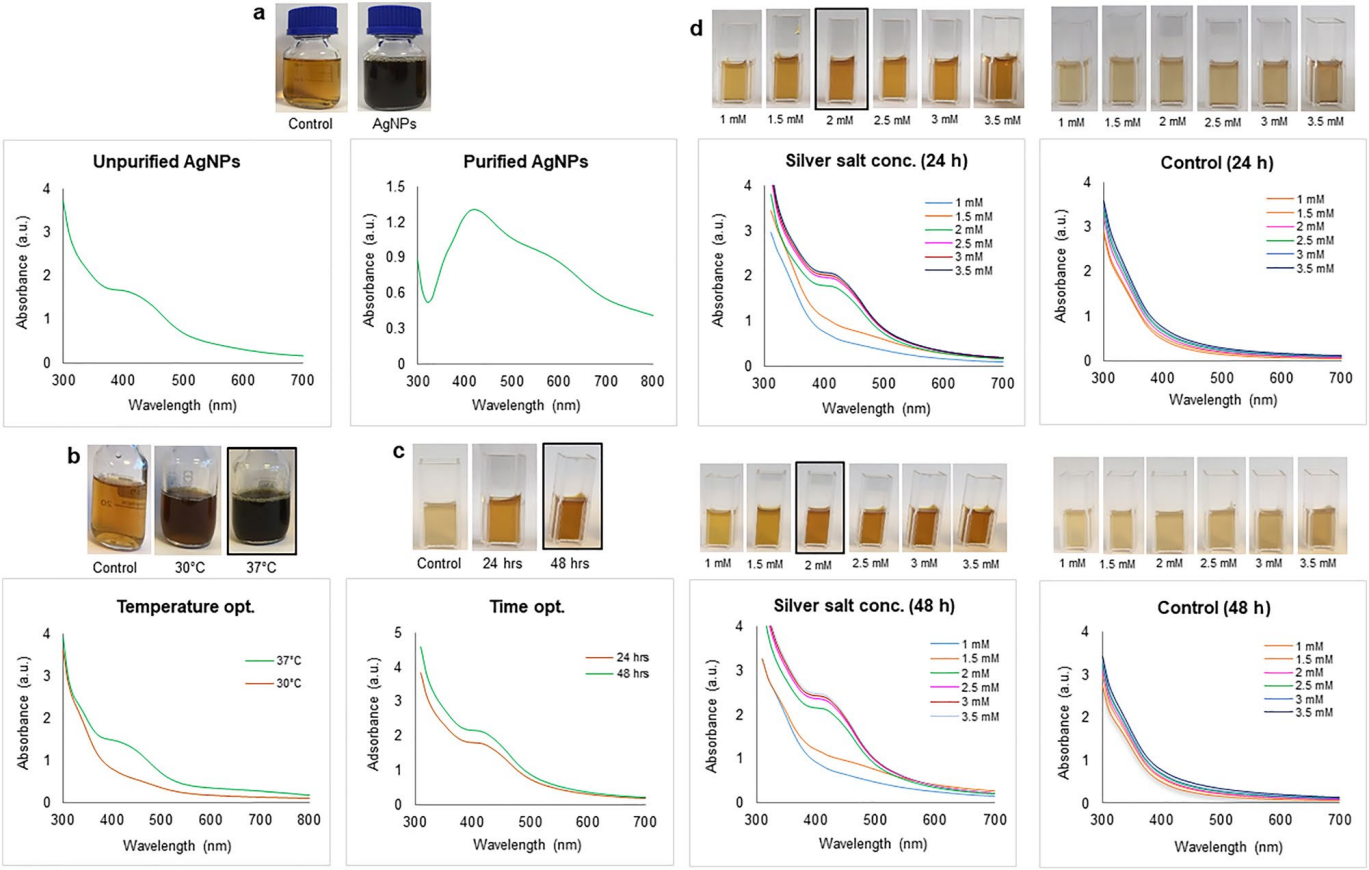
UV/Vis spectra (**a**) unpurified AgNPs in supernatant and purified AgNPs. Optimization studies based on UV/Vis spectral analysis (**b**) temperature, (**c**) time, and (**d**) silver salt concentration.

Supernatant derived from an overnight culture of *Cedecea* sp. was supplemented with 1 mM AgNO_3_ and used as a reducing medium for the production of AgNPs. After the incubation period, the supernatant showed visible colour change from pale yellow to deep brown, (Fig. 3a)^33^. To investigate the production of AgNPs quantitatively, UV–Vis spectrophotometer analysis was performed by scanning the reaction mixture in the range of 300–700 nm (Fig. 3a). The synthesis was confirmed by first directly scanning the reaction mixture after incubation period and then with purified nanoparticles suspension as well. The change in color is attributed to the surface plasmon resonance (SPR) property of AgNPs^34^. In addition, the maximum absorbance of the reaction mixture was absorbed in the range of 400 nm to 500 nm, which is specifically for the AgNPs^35^. Next, we attempted to optimize AgNPs production with respect to the amount of silver salt added, reaction temperature and incubation time. As shown in (Fig. 3b–d), optimal salt concentration was 2 mM, the optimal temperature was 37 °C, and the optimal incubation period was 48 h. Any further increase of these key parameters above the optimized values led to the disruption of UV–Vis peaks, indicating nanoparticle destabilization.

### Produced AgNPs are in size range of 10–40 nm, covered with biological material and highly stable

**Figure 4:**
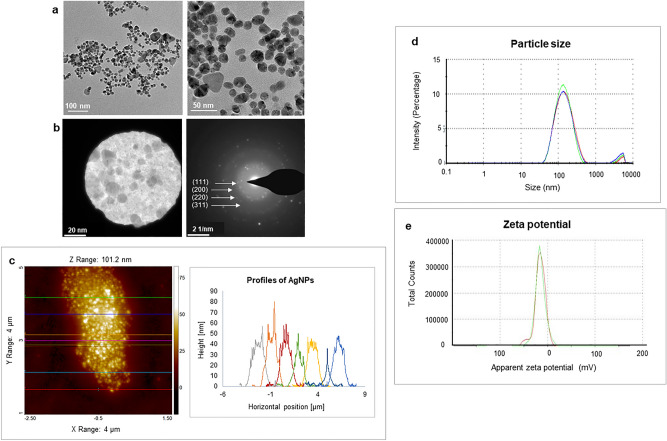
(**a**) Transmission electron microscopy images of produced AgNPs—nanoparticles shape and (**b**) selected area (electron) diffraction (SAED) with respective apertures. (**c**) AFM analysis of nanoparticles (**d**) Particles size distribution concerning the intensity of AgNPs and (**e**) zeta potential distribution showed the negative zeta potential value. The study was done in triplicates, and the results are an average of three analyses.

**Figure 5:**
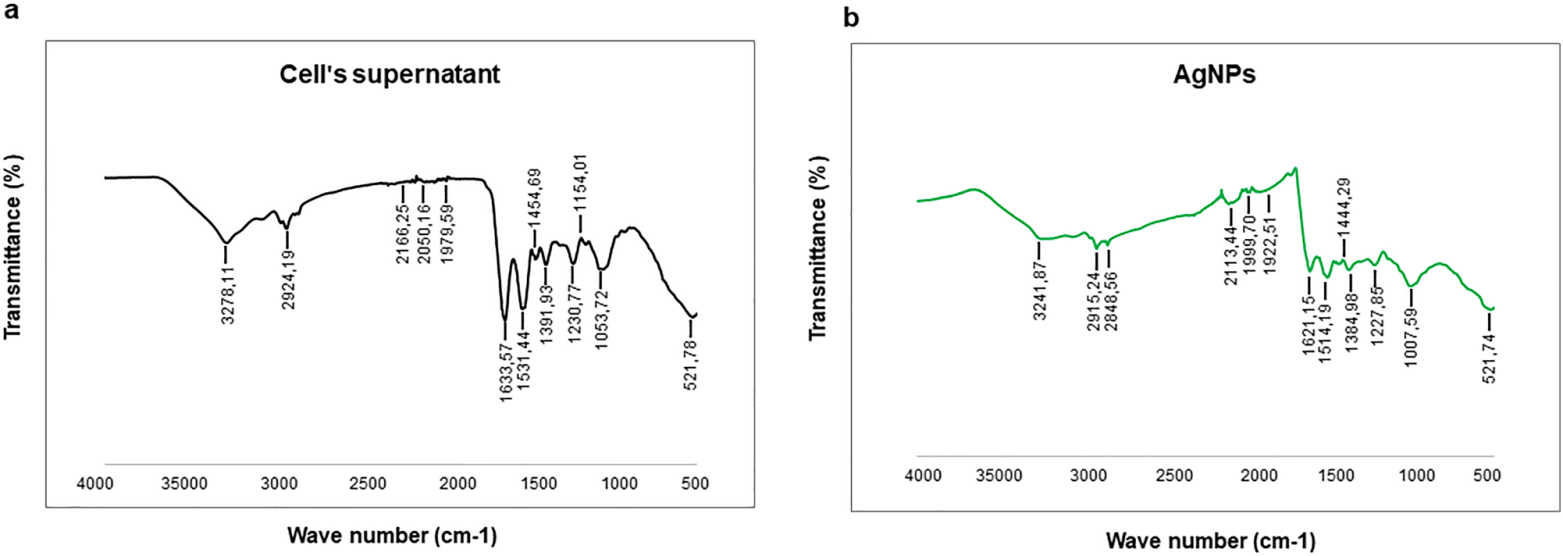
Fourier transform-infrared spectroscopy spectra of (**a**) Cell's supernatant and (**b**) AgNPs.

The morphology of produced AgNPs was studied by TEM, which showed that the nearly spherical shape is a dominant form, with some polydispersity of triangular and hexagonal shapes. The size of the AgNPs was determined to range from 10 to 40 nm (Fig. 4a). To analyze the phase of nanoparticles, selected area electron diffraction (SAED) analysis was carried out (Fig. 4b), which yielded results consistent with polycrystalline behavior^36^. The detected SAED pattern exhibited clear visible spots corresponding to the main reflection lattice planes of (111), (200), (220), and (311)^24,37^. The AFM analysis also showed the size of the nanoparticles falls in the range of 40–50 nm (Fig. 4c). Next, the size and surface charge of the produced AgNPs was examined by DLS. The average hydrodynamic diameter was determined to be 115.9 nm, with a polydispersity index (PDI) of 0.39 (Fig. 4d). The zeta potential value (surface charge) of the aqueous AgNPs solution at room temperature was found to be − 15.7 mV (Fig. 4e), which suggested that the NPs can be expected to be stable at neutral conditions^38^. To determine whether any functional groups or molecules from the bacterial supernatant attached to the surface of nanoparticles, FTIR measurements were conducted. These measurements revealed many functional groups specific for the bacterial cells' supernatant to be present on the nanoparticles' surface (Fig. 5a, b) (Table 1). For example, the peak observed at 3278.11 cm^−1^ for supernatant and 3241.87 cm^−1^ for AgNPs suggested they both share O–H and N–H stretching^39^. The asymmetric and symmetric C–H stretch was marked at 2924.19 cm^−1^ for cell and 2915.24 and 2848.56 cm^−1^ for AgNPs. The (carbonyl (C=O), amide I (N–H) and C=C groups were also found in both samples, in the region between 1600–1500 cm^−1^. The peaks identified at 1454.69 and 1391.93 cm^−1^ for the supernatant and 1444.29 cm^−1^ for AgNPs correspond to the C–H bending of COO^−^ or carboxylate groups. The overlapping of C–O, C–N, C–O–C and C–O–P stretching modes was also detected in both samples, at 1053.72 and 1007.59 cm^−1^ for the supernatant and AgNPs, respectively. Thus, the FTIR results indicate the presence of proteins, amino acids and other biomolecules originating from the supernatant on the surface of the produced AgNPs^39^. These active groups play a very important role in reducing metal salts and AgNPs stabilization.

**Table 1:** Fourier transform-infrared spectroscopy (FT-IR) spectra of bacterial cell's supernatant and AgNPs.

Type of Bond	Cells Wavenumber (cm^−1^)	AgNPs Wavenumber (cm^−1^)
O–H and N–H group	3278.11	3241.87
C–H group	2924.19	2915.24, 2848.56
Alkyne group	2166.25, 2050.16, 1079.59	2113.44, 1999.70, 1922.51
C=C, amide I (N–H), C=O groups	1633.57, 1531.44	1621.15, 1514.19
C–H bending, COO^−^ groups	1454.69, 1391.93	1444.29
Overlapping of C–O, C–N, C–O–C and C–O–P stretching modes	1053.72	1007.59
C–C deformation	521.78	521.74

**Figure 6:**
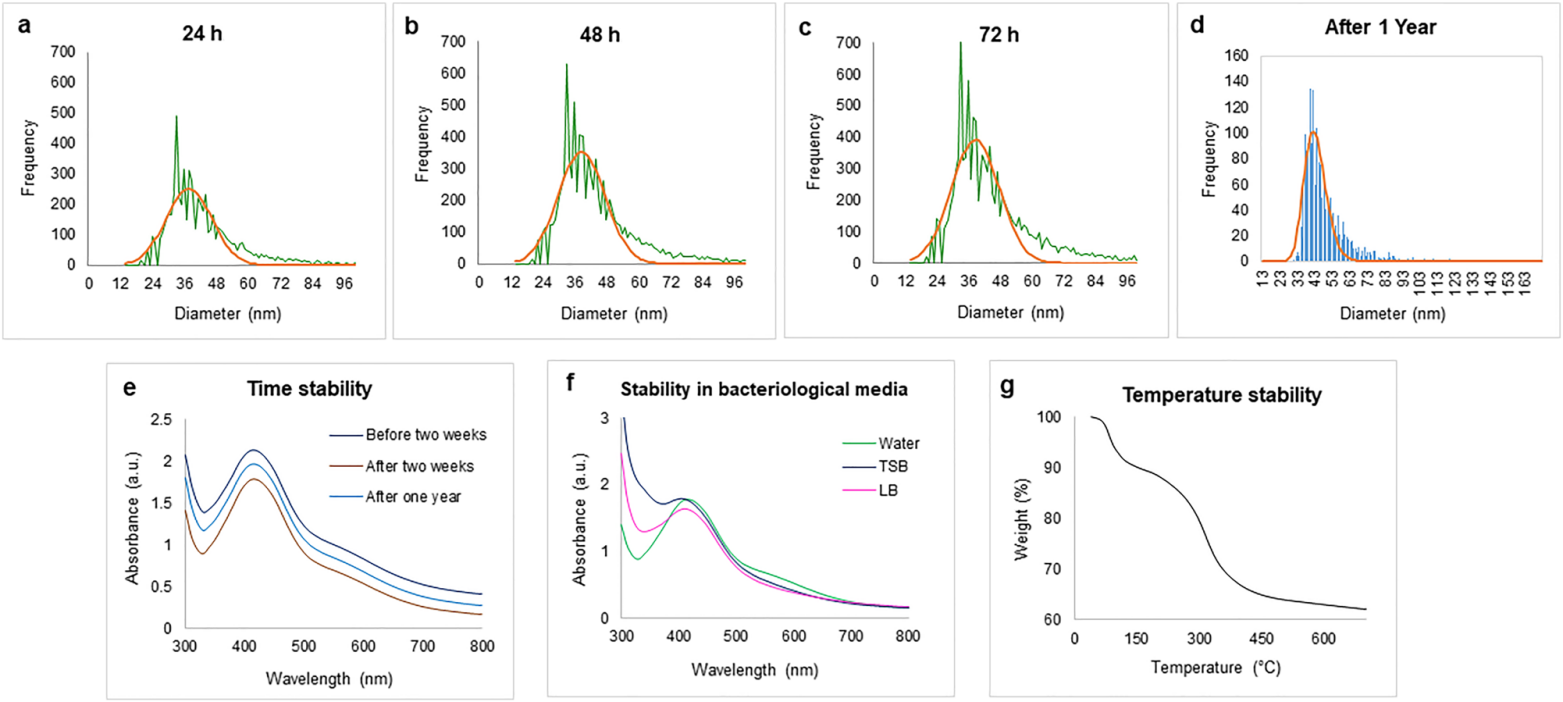
Inductively coupled plasma-mass spectrometry measurement of AgNPs, which shows the size distribution histogram. (**a**) ICP-MS measurement of AgNPs after 24 h, (**b**) 48 h, (**c**) 72 h (**d**) and one year of NPs formation (the green color shows the frequency of size distribution, and the orange line is the mean value). UV/Vis spectra for nanoparticles (**e**) time-dependent stability at a difference of two weeks and one-year (**f**) stability of AgNPs in bacteriological media. (**g**) Thermogravimetric analysis of NPsin the range of temperature 0–700 ℃.

To assess the efficiency of production by *Cedecea* sp., the concentration of AgNPs was determined by sp-ICP-MS (Fig. 6a–d). The measured total mass concentration was 1.31 µg/µl, with a negligible dissolved fraction (< 0.1 ppb). Next, we assessed short- and long-term nanoparticle stability. The measurements were carried out after 24 h, 48 h and one year. Our results indicated no significant difference in particle size, resulting in mean diameters from 49–55 nm, which indicated that the nanoparticles were stable up to one year^28^. The number of particles was not influenced by storage time, that no AgNPs dissolution or merging occurred even after one year. To confirm the aqueous stability, UV–Vis observations were made after two weeks of storing NPs in aqueous solution at room temperature and after one year at 4℃. As a result, no significant shift was observed, thus confirming the extreme aqueous stability of NPs (Fig. 6d). In addition, the AgNPs stability was tested in bacteriological media such as TSB and LB (Fig. 6e). The results indicated that the NPs are somewhat less stable in growth media^25^. For thermal stability, the TGA measurement was conducted. Figure 6f demonstrates the weight loss profiles with increasing temperature. Degradation of nanoparticles commenced above 400 °C, correlated to the loss of the organic phase materials adsorbed on the NPs surface^40^. A further increase in temperature up to 600 °C led to complete degradation of nanoparticles^41^.

### AgNPs produced in *Cedecea* sp. successfully inhibit bacterial biofilms

We first set out to determine the bacteriostatic and bactericidal activity of obtained AgNPs against planktonic bacteria employing measuring MIC and MBC of nanoparticles against *E. coli*, *P. aeruginosa*, *S. aureus* and *S. epidermidis* (Table 2). *E. coli* and *P. aeruginosa* were observed to be sensitive to AgNPs, with MIC values of 12.5 and 6.25 μg/ml and MBC values of 12.5 and 12.5 μg/ml, respectively. *S. aureus* and *S. epidermidis* were less sensitive to AgNPs, with both MIC and MBC values in the 100 μg/ml range (Table 2).

**Table 2:** Minimum inhibitory concentration (MIC) and minimum bactericidal concentration (MBC) of AgNPs against *E. coli*, *P. aeruginosa*, *S. epidermis* and *S. aureus*.

Bacterial strain	MIC (µg/ml)	MBC (µg/ml)
*E. coli*	12.5	12.5
*P. aeruginosa*	6.25	12.5
*S. epidermidis*	100	100
*S. aureus*	100	100

**Figure 7:**
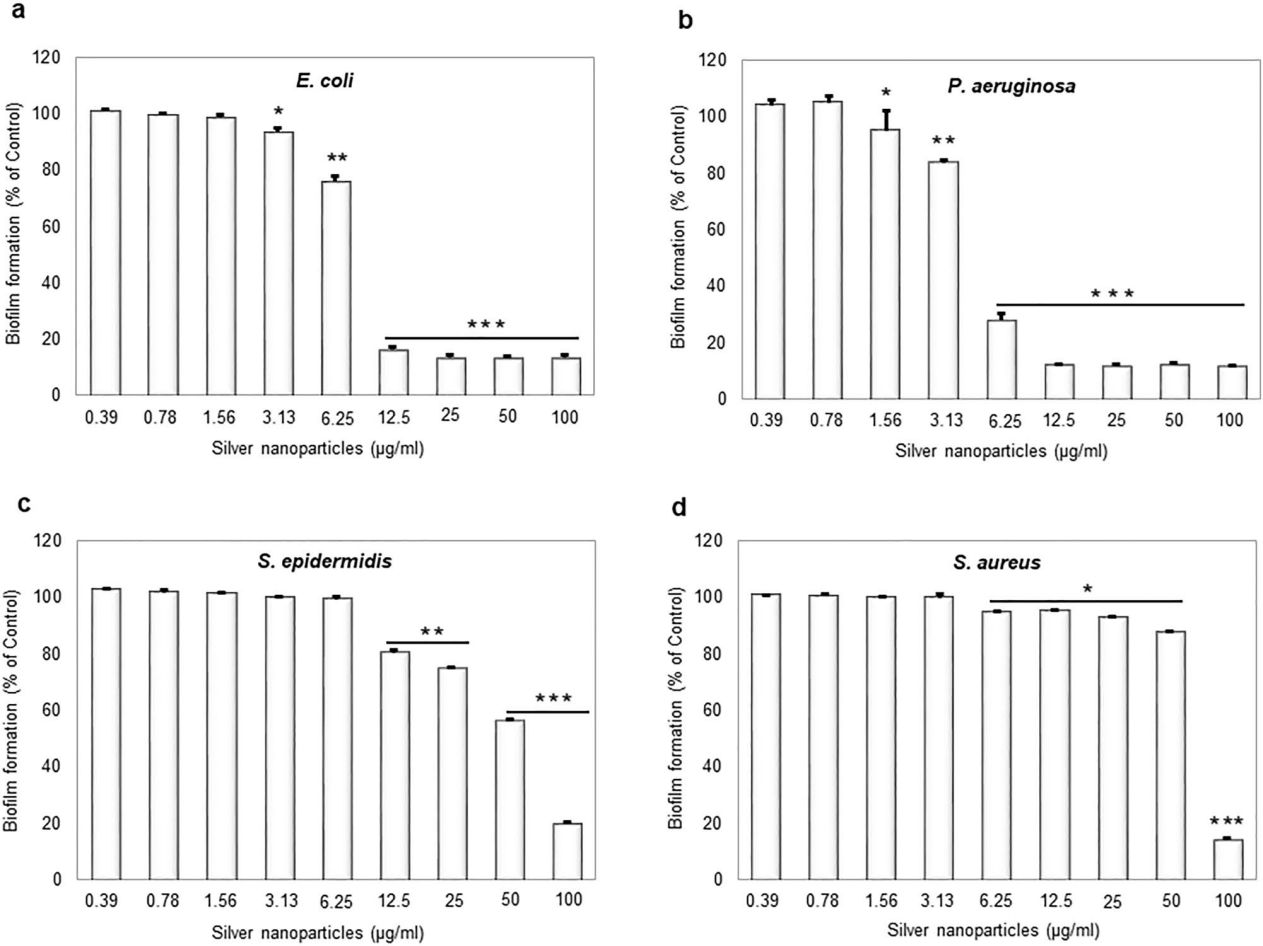
Effect of AgNPs on bacterial biofilm formation. (**a**) *E. coli*, (**b**) *P. aeruginosa*, (**c**) *S. epidermidis*, and (**d**) *S. aureus*. Data represent mean ± standard deviation error. *p < 0.05; **p < 0.005; ***p < 0.0001.

**Figure 8:**
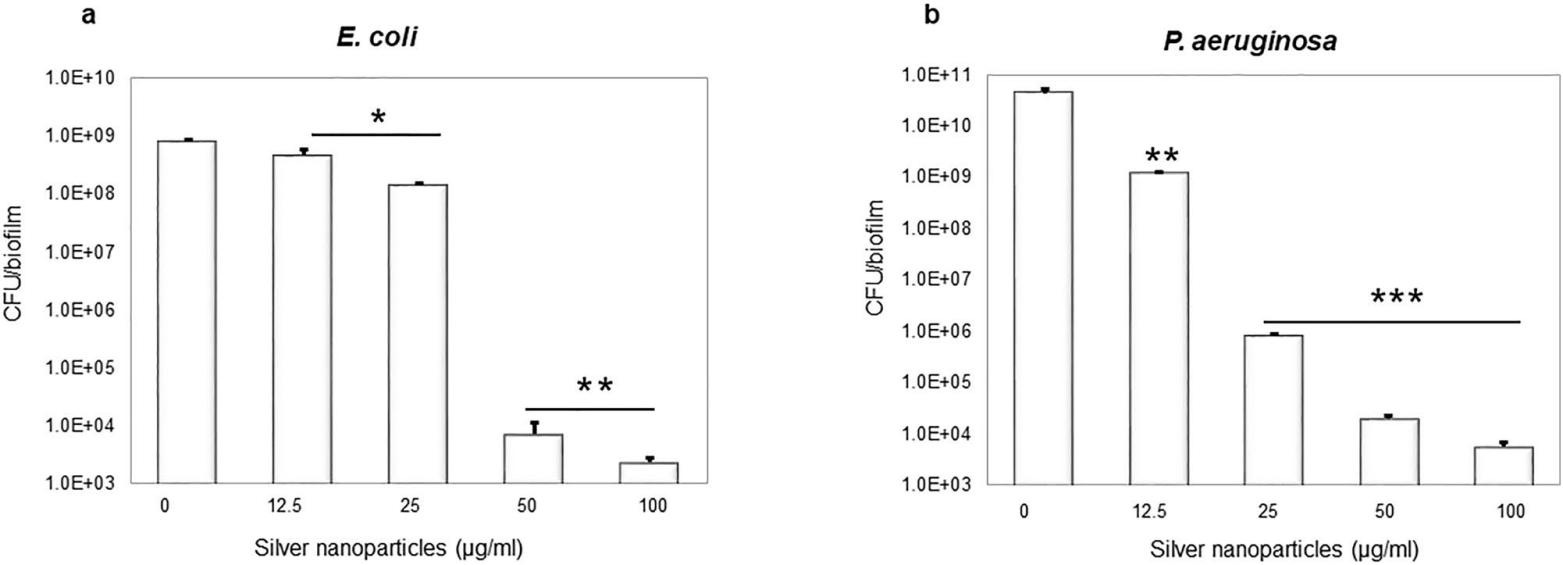
CFU count after treatment with AgNPs (**a**) *E. coli* and (**b**) *P. aeruginosa*. Data represent mean ± standard deviation error. *p < 0.05; **p < 0.005; ***p < 0.0001.

**Figure 9:**
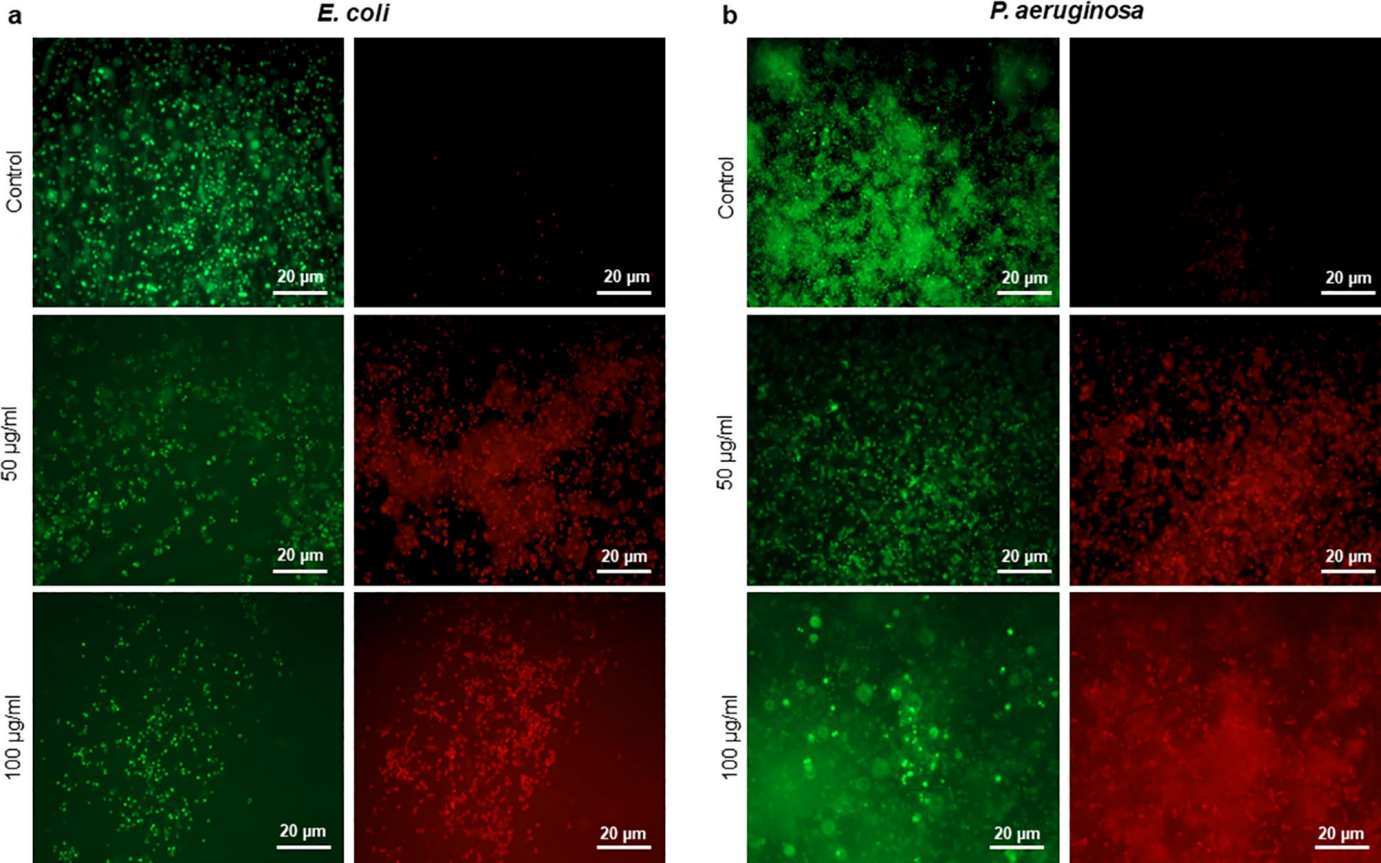
Live/dead staining image of biofilms observed by using fluorescence microscopy, (**a**) *E. coli* and (**b**) *P. aeruginosa*. Live cells stained green and dead cells stained red.

**Figure 10:**
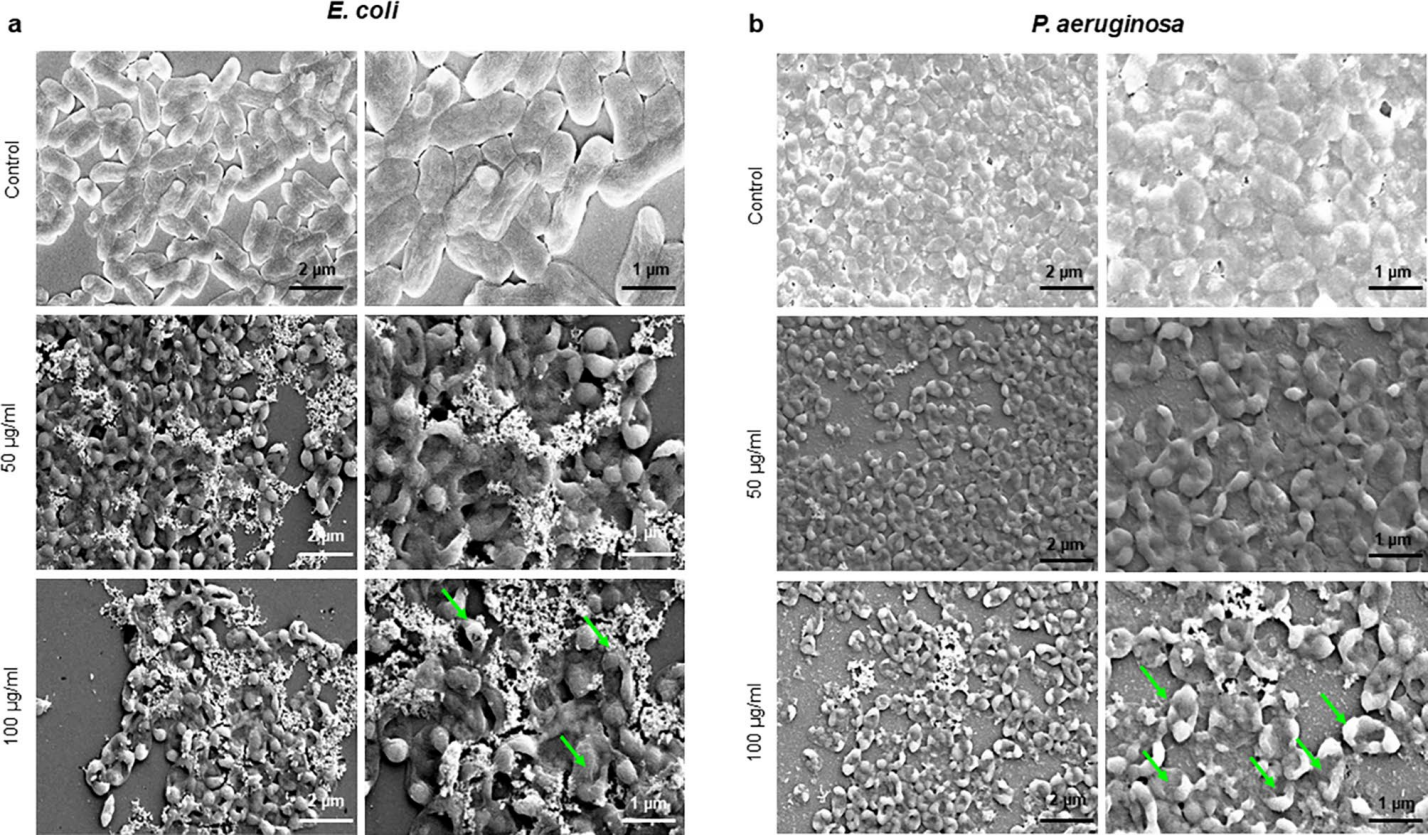
Scanning electron microscopy images of bacteria cells treated with different concentrations of AgNPs, (**a**) *E. coli*, and (**b**) *P. aeruginosa*. The green arrows indicate the cell membrane disruption due to the AgNPs effects.

Next, synthesized AgNPs were tested for inhibiting the formation of biofilm by the same bacterial species (Fig. 7). Concentration-dependent inhibition of biofilm formation was observed in all cases, stronger for *E. coli*, *P. aeruginosa* and weaker for *S. aureus* and *S. epidermidis* (Fig. 7). Inhibition of formation of *E. coli* and *P. aeruginosa* biofilms occurred at sub-MIC concentration (0.25 × MIC). Inhibition of biofilm formation can occur due to direct bactericidal effects or by inhibiting viable bacterial cells' attachment to the surface. To separate these two effects, next, we evaluated the direct bactericidal potential of AgNPs, by using mature, pre-formed biofilms of the two more sensitive species: *E. coli* and *P. aeruginosa* (Fig. 8)*.* CFU counts determined viability of biofilm cells following the AgNPs treatment counts determined viability of biofilm cells following the AgNPs treatment. A significant reduction in bacterial cell viability in both types of biofilm was observed with AgNPs concentrations above 25 μg/ml (Fig. 8). In order to confirm the viability data obtained by CFU counting, we used live/dead staining^42^. This method enables one to visualize individual live cells (stained green) and individual dead cells (stained red) under a fluorescence microscope (Fig. 9). These results confirmed a dramatic onset of the killing of bacterial cells at AgNPs concentrations above 25 μg/ml for both *E. coli* and *P. aeruginosa* biofilms. Given the known mechanism of killing bacterial cells by AgNPs, which involves extensive cell wall destruction and membrane protein inactivation^15^, bacterial cells' death should be accompanied by drastic morphological changes. To observe those, we used SEM (Fig. 10). A significant degree of morphological alteration was observed in AgNPs-treated biofilms. These included bacterial cells with visible membrane pores, cell lysis and leakage of intracellular content, clearly visible in both *E. coli* and *P. aeruginosa* biofilms. , The severity of these effects was correlated to the concentration of applied AgNPs for both bacterial species.

## Discussion

Our results indicate that *Cedecea* sp. can be a good host for the sustainable production of AgNPs with antibacterial potential. Optimizing the synthesis methodology, we concluded that maximum yields are obtained at 37 °C, after 48 h. of incubation and using 2 mM AgNO_3_ (Fig. 3). Further increases in any of these key parameters compromised nanoparticle stability. Nanoparticles produced by *Cedecea* sp. displayed several distinct advantages. The size of produced NPs within 10–40 nm offers a significant advantage in antibacterial applications because it favours the entry into target bacterial cells. The small size makes these nanoparticles also suitable for other medical applications, such as anticancer agents. ^43^.

FTIR confirmed the presence of a capping layer on the synthesized AgNPs. Although a slight shift in some peaks was observed (could be attributed to interactions between the biological compounds and AgNPs) our FTIR results agree with the published literature^25,44^ and corroborate the presence of a stable capping layer. The FTIR measurements confirmed that the capping layer's functional groups originated from the bacterial cells (Fig. 5). These groups are exposed on the NPs surface, where they contribute to NPs stability and play a role in the interaction of NPs with the surfaces of targeted bacteria. An important feature of the produced AgNPs is their inhibitory effect against biofilm-forming bacterial pathogens. The antibacterial and anti-biofilm activity of synthesized AgNPs was tested against both Gram-positive and Gram-negative bacterial strains. Recently, Shania et al. showed the S*phingobium* sp. MAH-11 mediated AgNPs MICs value for *E. coli* and *S. aureus* were 6.25 and 50 μg/mL, and the MBCs values were 25 and 100 μg/mL for *E. coli* and *S. aureus*, respectively^45^. Our AgNPs showed significant antimicrobial activity against the Gram-negative bacteria *E. coli* and *P. aeruginosa* at low concentrations. In contrast, the Gram-positive bacteria *S. epidermidis* and *S. aureus* were observed to be less sensitive^46^. This major difference in antibacterial effects against these two bacteria could be attributed to differences in bacterial surfaces. Gram-positive bacteria have a thick coating (20–80 nm) composed of negatively charged peptidoglycan, which might impede the penetration of AgNPs and attenuate the activity of silver ions, resulting in overall weaker antibacterial effects. In contrast, Gram-negative bacteria have a thinner cell envelope (8–12 nm) with negatively charged lipopolysaccharides, which are known to promote the adhesion of nanoparticles^47^. This makes the Gram-negative bacterial cells more susceptible to the treatment. Another contributing factor could be the overall electrical charge at the surface of bacterial cells. Surfaces of Gram-negative bacteria typically have a lower isoelectric point (> pH 2) compared to surfaces of Gram-positive bacterial (pH 3–4)^48^. It is hence possible that Gram-positive bacteria have a better capacity of neutralizing the charged functional groups in the AgNPs capping layer, and thus reducing their capacity of interaction with the target cells. Even though our AgNPs showed lower inhibition of bacterial growth for the Gram-positive bacteria, they still showed a strong inhibitory effect on biofilm formation, even at the sub-MIC concentrations. These results suggest that lower concentrations of AgNPs do not directly damage the bacterial cells; however, the release of silver ions after dissolution of the nanoparticles might be able to inhibit biofilm formation by altering gene expression or inhibiting quorum sensing within the biofilm^49^. It has been previously shown that the antibacterial activity of AgNPs is strongly correlated with their size. Smaller nanoparticles typically exhibit higher antimicrobial efficacy, due to easier internalization by bacterial cells. In the current study, the AgNPs were found to be in the size range between 10–40 nm, which corresponds well with the observed activity in damaging cell membrane and cellular components, clearly visible under SEM (Fig. 10). Our study's overall findings support a robust antibiofilm activity of our AgNPs against biofilms produced by *E. coli* and *P. aeruginosa*.

The most notable feature of AgNPs synthesized by *Cedecea* sp. is their stability. Owing to the capping layer formed by biological compounds from the bacterial supernatant, the AgNPs remained stable in water and in different growth media. For antimicrobial applications of these NPs, their stability is of the utmost importance. The SAED aperture results showed that our AgNPs are crystalline (Fig. 4b), their mean hydrodynamic diameter of 115 nm is stable, and they have a highly negative zeta potential value (Fig. 4e), all of which suggest high stability. The stability of AgNPs for up to one year was demonstrated by two independent methods (Fig. 6): the ICP-MS analysis and UV/Vis spectra recording. This remarkable stability of produced AgNPs most probably comes from the capping layer around the NPs surface, which is formed by the biological components secreted by *Cedecea* sp. Recently, Ragaa et al. also reported extreme stability of AgNPs produced by the cyanobacterium *Oscillatoria limnetica.* Stability was confirmed for up to 9 months, and the authors claimed that the capping agents helped in the long-term stabilization of the nanoparticles^44^. Our temperature stability analysis demonstrated the capping layer's involvement in stabilizing the nanoparticles (Fig. 6f)^28^. The organic phase materials adsorbed on the surface of the nanoparticle protected them up to 400 °C, and their gradual thermal destruction resulted in the complete disintegration of NPs at 600 °C.

## Conclusion

Our overall conclusion is that the growth supernatant of isolated *Cedecea* sp. offers a beneficial medium for synthesizing AgNPs for antibiofilm applications, particularly against Gram-negative pathogens. Functional groups from biomolecules present in the cell's supernatant form a stable capping layer on the produced NPs, contributing to their stability and promoting their interaction with target bacterial cells. With stability documented for up to one year, these AgNPs hold considerable potential for long-term use against biofilms of *E. coli*, *P. aeruginosa* and other Gram-negative pathogens.
